# Novel Tamoxifen Nanoformulations for Improving Breast Cancer Treatment: Old Wine in New Bottles

**DOI:** 10.3390/molecules25051182

**Published:** 2020-03-05

**Authors:** Candace M. Day, Shane M. Hickey, Yunmei Song, Sally E. Plush, Sanjay Garg

**Affiliations:** 1 School of Pharmacy and Medical Sciences, University of South Australia, Cancer Research Institute, North Terrace, 5000 Adelaide, SA, Australia; Candace.Day@mymail.unisa.edu.au (C.M.D.); Shane.Hickey@unisa.edu.au (S.M.H.); May.Song@unisa.edu.au (Y.S.); 2 Future Industry Institute, University of South Australia, 5095 Mawson Lakes, SA, Australia

**Keywords:** breast cancer, tamoxifen, nanotechnology, drug delivery systems, targeted therapy

## Abstract

Breast cancer (BC) is one of the leading causes of death from cancer in women; second only to lung cancer. Tamoxifen (TAM) is a hydrophobic anticancer agent and a selective estrogen modulator (SERM), approved by the FDA for hormone therapy of BC. Despite having striking efficacy in BC therapy, concerns regarding the dose-dependent carcinogenicity of TAM still persist, restricting its therapeutic applications. Nanotechnology has emerged as one of the most important strategies to solve the issue of TAM toxicity, owing to the ability of nano-enabled-formulations to deliver smaller concentrations of TAM to cancer cells, over a longer period of time. Various TAM-containing-nanosystems have been successfully fabricated to selectively deliver TAM to specific molecular targets found on tumour membranes, reducing unwanted toxic effects. This review begins with an outline of breast cancer, the current treatment options and a history of how TAM has been used as a combatant of BC. A detailed discussion of various nanoformulation strategies used to deliver lower doses of TAM selectively to breast tumours will then follow. Finally, a commentary on future perspectives of TAM being employed as a targeting vector, to guide the delivery of other therapeutic and diagnostic agents selectively to breast tumours will be presented.

## 1. Introduction

Breast cancer (BC), defined as the uncontrolled growth and rapid proliferation of breast cells, originating from the lobules or ducts, to other regions in the body [1]. BC is the second leading cause of cancer-related women fatality; trailing only lung cancer [2]. It is the most commonly diagnosed cancer in European women, with approximately 350,000 new cases and 130,000 deaths reported each year [3]. In 2016, a total of 1,685,210 new cases of BC were expected in the US and approximately 595,690 succumbed to this insidious disease [2]. The majority of new BC cases is reported in women over 50 years of age [1,2,3,4]; with 1 in 77 individuals at risk of fatality from BC by their 85th birthday [4].

The early stages of BC often present as a firm and thickened “lump” [5,6]. In later and more advanced stages, there is a change in the appearance of the breasts as evidenced by the development of swelling bumps, unexplained itchiness, redness and painful sensations [6]. BC is usually diagnosed using the *Triple Approach*, which consists of taking an extensive family history; an examination of the breast using imaging techniques such as magnetic resonance imaging, mammogram or ultrasound; and a biopsy of the breast tissue for further examination [7,8]. Treatment options usually depend on the cancer type and stage of cancer progression, whilst potential side effects are also considered. Treatment options include surgery, radiotherapy, hormone therapy, and standard chemotherapy and personalised targeted therapy with a combinatorial approach often being selected. For example, chemotherapy or radiotherapy agents are often given to patients after they have undergone surgery to destroy any remaining cancers [7,8,9].

For early-stage BC, breast-conserving therapy (including surgery and breast irradiation), mastectomy and lymph node dissections are the treatment options typically pursued [7,8,9]. The choice of treatments can be influenced by various factors, including socioeconomic status, treatment logistic feasibility, tumour characteristics and accessibility of treatment facilities [9,10]. Depending on the tumour types and hormone receptor status, chemotherapy and up to a five-year hormone therapy course may be included in the eventual treatment regime [8,9,10].

### BC Targeting Therapy

Even though chemotherapy is still used as the clinical standard, targeted therapy which uses drugs to block cancer cells from growing and spreading is attracting increased attention from experts in the field [11,12,13]. Traditional chemotherapy drugs are non-selective; they migrate to almost all parts of the body via the bloodstream [11], interfering with cellular DNA synthesis and destroying both rapidly dividing cancerous and healthy cells. This results in many of the negative side effects associated with chemotherapy strongly affecting long-term quality of life [14]. Around 36 different side effects are commonly reported, including fatigue, nausea, hair-loss and memory impairment [14]. More dangerously, a significantly higher risk of developing a second non-breast primary cancer has been reported in older patients receiving chemotherapy [15]. In contrast, targeted therapy focuses on delivering small therapeutic molecules and immunology materials, such as antibodies, to specific molecular targets located on tumour membranes [12,13]. Therefore, targeted therapy offers a more site-directed treatment option for BC and will inherently result in fewer off-target effects. Understanding the modes of action employed by the aforementioned molecular targets over-expressed on breast tumours and their induction of cancer growth is essential, especially in the finding of potent antagonists to reduce the influence of these receptors to promote cancer apoptosis.

Endocrine receptors (EnRs) and human epidermal growth factor receptor 2 (HER2) receptors are some of the most common molecular targets used in BC targeted therapies [16]. EnRs can be divided into estrogen receptors (ERs) and progesterone receptors (PRs). Approximately 80% of BC cases develop in response to estrogen; hence, they are classified as *Estrogen Receptor Positive* (ER+) BC [16,17,18,19]. ERs are further divided into two subclasses: ERα and ERβ; each exhibiting different cellular locations and biological functions [16,17,18,19]. ERα is mainly found on mammary glands and the uterus and its activation is responsible for the proliferation of BC, whilst ERβ is expressed predominantly on the prostate [16,17]. Currently, only ERα is used as a drug target because the exact role of ERβ in BC is still unknown [17]. The expression of progesterone is conditional on estrogen expression; for 65% of ER+ BC cases the level of progesterone will also increase [18]. ER/PR positive BC patients can benefit significantly by using ER antagonists or selective estrogen receptor modulators (SERMs) to selectively block the action of estrogen [20]. About 15% of all primary BC cases are HER2 positive. Compared to ER/PR positive BC, patients diagnosed with HER2-positive BC have a shorter survival median and tend to develop relapses; therefore, HER2 expression should be tested for in every case of BC [20,21,22]. Through anti-HER2 therapies, HER2 can be successfully targeted using monoclonal antibodies including trastuzumab and pertuzumab; tyrosine kinase inhibitors such as lapatinib and neratinib; or antibody-drug conjugate such as ado-trastuzumab emtansine [21,22,23]. Instances where BC develops without the support of ER, PR and HER2 is referred to as *Triple-Negative Breast Cancer* (TNBC) [24]. TNBC is responsible for approximately 25%–30% of cancer cases in patients under 50 years old [24]. Targeted therapy has so far failed to effectively treat TNBC, and consequently clinicians elect for cytotoxic chemotherapy treatment for this cancer type [24].

Given ER is the most important molecular target in BC targeted therapy, the use of selective estrogen receptor modulators (SERMs) which block the effects of endogenous estrogen in breast tissues thereby competing with estrogen for ER binding, has been acknowledged as one of the most effective strategies [20]. Tamoxifen (TAM) is the most well-known SERMs, and acts by promoting cancer cell death by down-regulating the action of ERs.

## 2. TAM as a Gold Standard in BC Therapy

**Figure 1:**
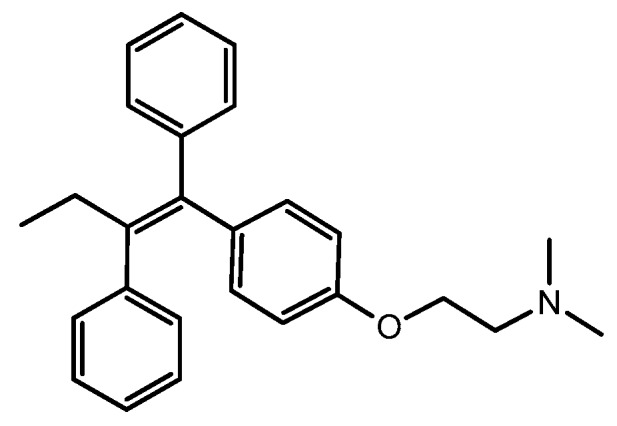
Tamoxifen (TAM) (PubChem CID: 2733526).

TAM, chemical structure demonstrated in Figure 1, was discovered by Richardson in 1962 in the British chemical group ICI facility, as a potential morning-after pill, owing to its effective post-coital contraceptive activity in rats [25,26,27,28,29]. However, its use as a contraceptive was swiftly ceased after preliminary human trials concluded that TAM actually induced ovulation [30,31]. It was later discovered that TAM could both act as an agonist and antagonist to ER, depending on the type of tissue and organ [30]. Unlike its agonistic effect on the reproduction tract, TAM was found to compete with the binding of estrogen to ERα on mammary glands, ultimately perturbing the ERα signaling pathway [25,32]. In 1973, TAM was classified as a nonsteroidal SERM; the mode of action of TAM is based on its ability to compete with estrogen and estradiol for the binding to ERs in breast tissues [33]. The half-life of TAM is approximately five to seven days with 65% of the dose hepatically eliminated over two weeks [25].

The anticancer ability of TAM against MCF-7 ER+ BC cell lines was confirmed by Lippman and Bolan [34]. The inhibition of BC cells at the G1 phase by TAM has also been demonstrated with work by Perry et al. [35]. As estrogen overproduction leads to tumour growth, indicated by the overexpression of ERs on tumour membrane, TAM antagonist effects against ERs will hinder DNA synthesis and cellular responsiveness of cancer cells to estrogenic stimulatory effects, thereby promoting cell death [33,34,35]. Furthermore, TAM prevents the growth of tumours by upregulating the tumour-inhibiting transforming growth factor B (TGFb) and downregulating the tumour-stimulating insulin-like growth factor 1 (IGF-1) [33,34,35]. As a result, after its potential application to treat metastatic BC was discovered in 1973, TAM once again was brought back into the spotlight [36]. Consequently, the use of TAM (branded as Nolvadex] for the treatment of metastatic BC was approved in 1977 [36].

A real breakthrough application occurred when TAM was used as an adjuvant therapy in treating BC [34,37,38]. While there had been fears that long-term adjuvant TAM would eventually give rise to premature drug resistance [31,37], several studies in that late 1970s revealed that a three-year TAM adjuvant therapy approach is significantly superior to less TAM-intensive strategies [38,39,40].

In 1977 the first long-term TAM adjuvant therapy to chemotherapy (five years) was conducted in node-positive patients [41]. This pilot study reported no unusual adverse effects driving the five-year TAM adjuvant therapy, with plasma concentration of TAM and its metabolites remaining stable throughout the duration of the trial and patient survival rates improving. Following the trial, many participants chose to extend the therapy to 14 years [41,42]. Simultaneously, a Scottish trial also reported that a five-year TAM adjuvant therapy holds a significant survival advantage for BC patients, compared to a TAM-free treatment [43]. It is now acknowledged that a ten-year TAM adjuvant therapy strategy is even more advantageous than a five-year adjuvant therapy, with a 50% decrease in mortality, compared to that of 30% associated with the five-year regimen [44]. Overall, these studies have concluded that long-term administration of up to five years or longer might be the best clinical strategy for TAM adjuvant therapy. Furthermore, in 1998 TAM was approved as a preventative treatment for women deemed high-risk for developing BC [45]. After almost 40 years from when TAM was first discovered as an ultimately unsuccessful contraceptive, patients are administered TAM as an effective chemo-preventative drug for BC therapy [46].

Unfortunately, a link between TAM and endometrial cancer was observed through various trials during 1988 and 1994 [47]. However, these trials also suggested that TAM becomes carcinogenic only when high doses of TAM are administrated to patients; well above the conventional dose of 20 mg/day [47,48,49]. TAM has also been found to induce liver cancer in rats [50], but interestingly, the similar long-term carcinogenicity results were not replicated in mice which are the standard animal model used during earlier stages of drug development [50]. However, the fact that millions of women have been treated with TAM since 1977, without the increased human hepatocellular carcinoma incidence being reported suggests that the finding of liver cancer in rat models receiving TAM has minimal relevance to humans and BC therapy.

## 3. Nano-Enabled-Formulations Containing TAM

Even though TAM is widely used in BC treatment, concerns remain regarding TAM-induced endometrial and liver cancer which has stymied its long-term therapeutic use [51]. As previously mentioned, most side effects observed with TAM are dose and concentration dependent. Therefore, it is reasonable to conclude that low dosing is the key solution in balancing the benefits and side effects of TAM. Nanotechnology is well-known as one of the most successful strategies to enhance the therapeutic efficacy, safety profile and accurate delivery of drugs [52]. Exploiting the advantageous properties of nanomaterials, many nano-enabled systems have been fabricated to carry TAM molecules and deliver them specifically to breast tumours, with high accuracy and minimal off-target side effects [51,52]. The incorporation of TAM into nanoscaled drug-delivery systems has delivered TAM molecules a much lower concentration over a prolonged period of time, hence, greatly reducing its risk of dose-dependent toxicity [51].

Desired therapeutic efficacy can only be achieved if a required concentration of an active drug reaches its target site [52]. The optimum concentration in which TAM can be effective may be negatively reduced, mainly due to the protective mechanism of the body, or the presence of macrophages from the reticuloendothelial system and tumor-associated macrophages which destroy TAM or foreign molecules [53]. Therefore, besides the ability to deliver TAM at a smaller and safer dose to tumours, nano-enabled-formulations acting as carriers can protect hydrophobic TAM molecules from degradation by macrophages during their transportation within the blood to prolong their circulation time, ensuring a required concentration of TAM can reach the tumour’s site [51,53]. This was evident from previous studies reporting that about 1% to 5% of nanoformulated drugs can accumulate in a target region, compared to less than 0.01% of an injected dose of native drugs [54].

**Figure 2:**
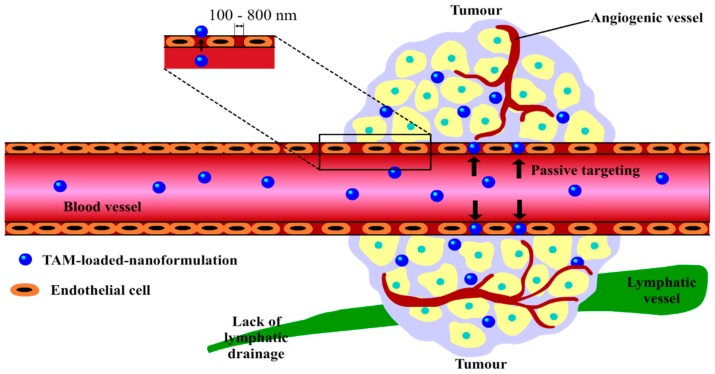
Passive delivery of TAM-loaded-nanosystems to tumours via the enhanced permeability and retention (EPR) effect. The explanation for this concept is that, as tumour cells grow quickly, their needs for nutrients and oxygen supply also increase rapidly, simulating the production of new tumour blood vessels with abnormal architectures, or angiogenic blood vessels. These rapidly formed and premature tumour vessels are made up of poorly aligned endothelial cells with large gaps (usually about 100 to 800 nm) between them, allowing TAM-loaded-nanoformulations with appropriate sizes to enter [54,55,56]. In addition to this, these nanosystems are retained inside tumour tissues for days and even weeks, due to the lack of effective lymphatic drainage, allowing TAM molecules sufficient time to be released from carriers and take effect [54,55,56]. Image adapted from Dai et al. [55].

A key requirement of nanoformualtions suitable for use in cancer drug delivery is the molecular scale, often 100 to 800 nm, allowing them to exploit the tumour environment for selective drug delivery via the enhanced permeability and retention (EPR) effect [54,55,56]. This effect is demonstrated in Figure 2; as TAM-loaded-nanoformulations are of nanosizes, they are able to penetrate into the leaky blood vessels surrounding cancer cells more easily compared to larger native TAM molecules, increasing passive cellular uptake of TAM-nanosystems by tumours, consequently enhancing the delivery of TAM [54,55,56].

Many attempts to fabricate TAM-loaded-nanoformulations from various nanomaterials have been made, with varying degrees of success, utilising the therapeutic effects of the multifunctional drug TAM. This review will discuss recent advances in the synthesis of different nano-carriers to deliver TAM to breast tumours, with specific focuses on liposomes, micelles and other types of nanoparticles.

### 3.1. Liposomes

In 1995, the very first nanomedicine approved by the FDA was Doxil—a liposomal pharmaceutical product containing doxorubicin (DOX)—used to passively target cancer [54]. Since the success of Doxil, liposome-based products started to gain the attention of researchers in the fields of nanotechnology and drug delivery. Liposomes are spherical and closed bi-layer phospholipid systems which can be used to encapsulate both hydrophilic agents within their aqueous core [52,54], and lipophilic drugs such as TAM within their lipid bi-layer. By the housing of TAM within the phospholipid membrane, smaller concentrations of TAM molecules can be protected from macrophage-induced degradation, prolonging their systemic circulatory half-life and enhancing the chance of TAM molecules to arrive at their target site [51,57,58].

Stable TAM-loaded liposomal formulations have been synthesised from soya phosphatidylcholine (SPC), cholesterol (CH) and Span 20, using a thin lipid film hydration method [59]. This system achieved sustained release of TAM, with 50% drug molecules released within three hours, and 95% of drug released after 30 h, as evidenced using in vitro release experiments. In another study conducted by Lin et al., a cationic liposome-PEG-polyethylenimine (PEI) complex (LPPC) was used as a nanocarrier, designed specifically for the local delivery and transdermal release of TAM [60]. The LPPC/TAM demonstrated a dramatic increase of activity against all BC cases, especially in ER+ BC cells. Besides the efficacy of LPPC/TAM complexion to most breast cancer cell lines, the local administration of LPPC/TAM did not induce skin or organ injury, suggesting their significant potential as a transdermal treatment for breast cancer [60].

**Figure 3:**
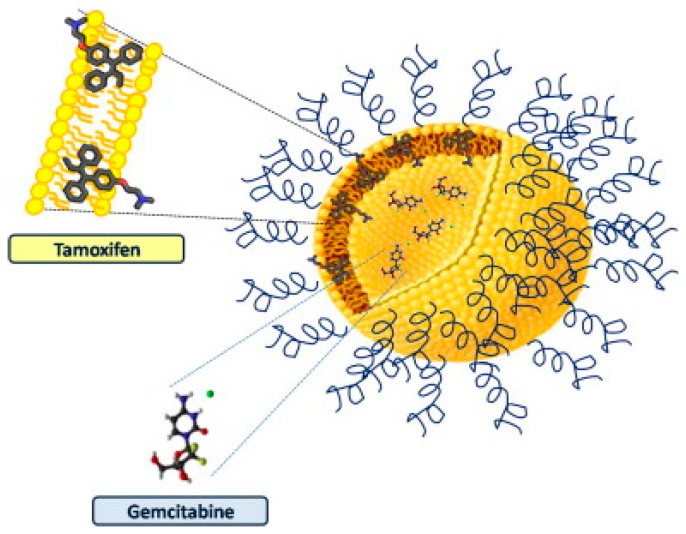
Graphic illustration of TAM/ gemcitabine (GEM) liposome and their localisation inside the multidrug (MD) carrier. TAM was incorporated into the lipid bilayer of liposomes; while GEM molecules were loaded inside the hydrophilic central core of liposomes, allowing the co-loading of two therapeutic agents to occur. Image taken from Cosco et al. [61].

Exploiting the structure of unique physicochemical and technological properties of liposomal lipid composition, liposomes can also be employed as a multidrug carrier (MDC), for the co-delivery of TAM and other therapeutic agents. For instance, Cosco and colleagues fabricated a liposomal system containing both TAM and gemcitabine (GEM), as illustrated in Figure 3, using various phospholipids such as 1,2-dipalmitoyl-s*n*-glycero-3-phospocholine monohydrate (DPPC), dimyristoyl phosphatidylglycerol (DMPG), *N*-(carbonyl-methoxypolyethylene glycol-2000)-1,2-distearoyl-s*n*-glycero-3-phosphoethanolamine (DSPE-MPEG 2000), fabricated via a thin-layer evaporation technique and extrusion process [61]. Following the investigation of their antitumor activity on MCF-7 and T47D cell lines, liposomal MDC demonstrated superior results in cell viability compared to each single drug in their native form (95% vs. 70% cell reduction in MCF-7, 50% vs. 40% cell reduction in T47D), suggesting this liposomal system is a promising tool for the compatible co-delivery of TAM with different chemo-agents.

Recent innovations of mentioned TAM-loaded liposomes and their advantages in the treatment of BC from 2008–2019 are summarised in Table 1, with respect to their compositions, physiochemical features as well as the claimed advantages.

### 3.2. Micelles

Unlike liposomes that are bi-layer systems made up of phospholipid units, micelles are single-layer amphiphilic self-assembly architectures, formed by repeating units of surfactant molecules [52,66,67]. At low concentrations, surfactant molecules exist separately; however, when their concentration is increased, they aggregate to form micelles, within a narrow concentration range referred to as the critical micelle concentration (CMC) [66,67]. Micelles can accommodate hydrophobic cargoes such as TAM in their cores, while the hydrophilic corona protects them from being degraded by surrounding environment [51,52]. Being small in size, usually in the 20–80 nm range, micelles can easily penetrate into the leaky vasculature surrounding tumours and are subsequently retained there for longer periods of time owing to EPR effect [66,67]. Even though micelles demonstrate some undeniable advances in the delivery of TAM; compared to liposomes, they have smaller drug-loading capacity and lower stability, due to the reversibility of their monomer subunits [67]. However, micelles generally have less toxicity and can be eliminated more easily from the body through renal filtration. Moreover, the aqueous solubility of hydrophobic drugs can be increased up to 500-fold once they are encapsulated inside polymeric micelles [68].

Gao et al., (2002) have developed TAM-loaded micelles from PEG 5000 modified with distearoyl-phosphatidylethanolamine (PEG5000-PE) for the enhanced tumour uptake of TAM [69,70]. These micelles were able to incorporate up to 20 wt% of TAM, and demonstrated increased drug accumulation into C57BL/6J tumour bearing mice, especially through tail vein injection [69,70]. Another TAM-loaded micellar system was synthesised from poly(latic-co-glycolic acid) PLGA-PEG diblock copolymers, was able to enhance bioavailability of TAM in the epidermis 3.5-times compared with native TAM. The antitumour activity of the system against MCF-7 cell lines was also significantly enhanced [71].

**Figure 4:**
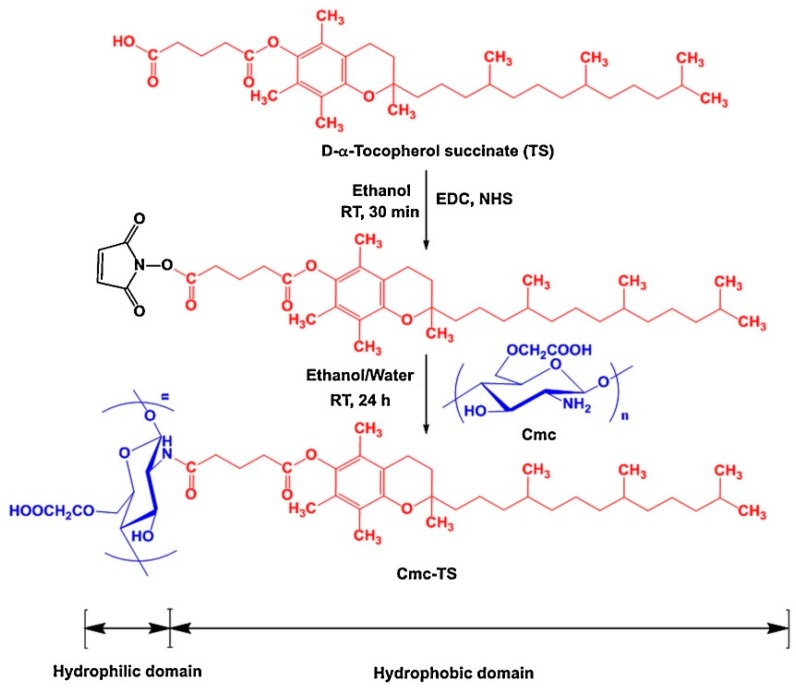
Schematic illustration of the synthesis of α-tocopherol succinate-g-carboxymethyl-chitosan via carbodiimide chemistry. The carboxyl group of α-tocopherol succinate was conjugated with the amine group of chitosan (Cmc) of low molecular weights, with 1-Ethyl-3-(3-dimethylaminopropyl)carbodiimide (EDC) and N-Hydroxysuccinimide (NHS) employed as coupling agents. In the final step, Cmc-TS were obtained by lyophilization. Image taken from Jena and Sangamwar [72].

Recently, novel nanosized self-assembled core shell structured micelles prepared from low molecular weight carboxymethyl chitosan and α-tocopherol succinate (TS) from a novel co-solvent evaporation technique, as demonstrated in Figure 4. The system achieved a maximal TAM loading up to 8.08 ± 0.98%. The stability of the system was demonstrated using in vitro release experiments in simulated gastric and intestinal fluid, with a pH dependent release profile observed. Oral absorption was also demonstrated in vivo with a 1.9-fold increase in bioavailability observed, when compared to free drug molecules [72].

Another novel micellar TAM system derived from palmitic acid and chitosan co-polymers, was recently fabricated and evaluated by Thotakura et al. [73]. The nanocarriers were able to substantially incorporate TAM molecules, and provide a controlled release of TAM. More importantly, these nanosystems significantly enhanced the cytotoxicity of TAM against MCF-7 cancer cells by almost double due to the enhanced cellular uptake. The intravenous formulations are five times more haemo-compatible, compared to native drug [73]. Recent innovations of TAM-loaded-micelles and their advantages in the treatment of BC from 2008–2019, are summarised in Table 2, with respect to their compositions, physiochemical features as well as the claimed advantages.

### 3.3. Other Nanoparticles

The most commonly employed nanoparticles (NPs) in cancer drug delivery are polymeric nano particles (PNPs)—fabricated primarily from poly d,l-lactic-co-glycolide (PLGA), starch and chitosan—which exhibit excellent safety profiles, biocompatibility, biodegradability and cost-effectiveness [51,75]. TAM molecules are held within the PNPs by hydrogen bonding and lipophilic interactions to give stable drug-polymer conjugations which protect TAM degradation [76]. Another TAM-loaded NP based on PLGA has been prepared and evaluated for its anticancer efficacy against MCF-7 cells [77]. TAM-loaded NPs may be suitable for BC treatment as they are able to obtain high drug loading, sustained release kinetics and high cellular uptake by MCF-7 cell lines in vitro. Ravikumara and colleagues were also able to improve the antitumour efficacy of TAM when tested on MCF-7 cell lines, as biodegradable poly(d,l) lactic acid was employed to fabricate TAM-loaded-polymeric-NPs [78].

In addition to PNPs, TAM-loaded solid lipid nanoparticles (SLNs) have recently been utilised as another approach to tackle BC. SLNs consist of solid lipid cores inside which TAM molecules can be dispersed, and are coated with a surfactant layer on the surface, preventing particle aggregation [52]. For example, TAM-loaded SLNs were found to promote apoptosis on MCF-7 and MDA-MB231 cells, similar to that of free TAM. Interestingly, the TAM-loaded SLNs were able to obtain a more prolonged release, suggesting their suitability for use as a controlled release drug delivery system (DDS) in BC, while reducing the hepatotoxicity associated with the free drug [79]. Another recent study demonstrated that TAM-loaded SLN formulations could be effective for overcoming TAM resistance in BC therapy, through their induction of decreased cell viability of MCF7 and MCF-7 TAM-resistant cell lines [80].

Jain et al. has investigated the potentials of TAM-loaded liquid crystalline nanoparticles (TAM-LCNPs) [81]. Hexagonal TAM-loaded glyceryl monooleate and TAM-loaded phytantriol-based LCNPs (PLCNPs) exhibit small particle sizes with a narrow distribution, high stability in the gastrointestinal tract and a sustained drug release profile. Both TAM-loaded LCNPs were able to displace 7.3 to 10-fold increments in IC_50_ values respectively, compared to their native counterpart, indicating significantly enhanced antiproliferative efficacy [81].

Another commonly employed type of TAM-loaded NP is metallic nanoparticles (MNs). Recently, TAM-loaded chitosan-coated silver NPs (Tam-CS-AgNPs) were explored as delivery systems of TAM to MCF-7 human BC cells [82]. Treatment of cancer with TAM-CS-AgNPs for 24 h induced cancer cell death and tumour membrane leakage, in a dose-dependent manner. The apoptotic effects of TAM-CS-AgNPs were attributed to their activation of caspase-3 and DNA nuclear fragmentation, rendering them as promising anticancer DDS candidates [82]. The use of magnetic MNs as TAM carriers in cancer delivery has also become popular. For instance, TAM-loaded-tyrosine (Tyr) modified Fe_3_O_4_ magnetic NPs (F@Tyr@TAM, where @ means incorporated) were synthesised and evaluated for biocompatibility, loading capacity, release profile, and anticancer activity on MCF-7 cell lines [83]. Haemolysis testing and MTT assays of F@Tyr@TMX NPs studied on MCF-7 cell lines indicated that the toxicity of bare Fe_3_O_4_ magnetic nanoparticles and F@Tyr@TAM are suitable for the delivery of TAM in BC therapy.

Protein-based nanoparticles have also played a vital role as drug carriers, owing to their promising in vitro results and their use with some anticancer agents. To overcome poor solubility of hydrophobic compounds like TAM in aqueous solution which hinders their delivery to breast tumours, highly water-soluble proteins with multiple binding sites with different affinity with many lipophilic compounds, such as serum albumins, are also employed as their nano-carriers [84,85,86,87,88,89,90,91,92]. For example, thiol coated alginate-albumin nanoparticles were constructed using a coacervation method to deliver TAM to cancer cells. The TAM-loaded-NPs demonstrated anticancer efficacy when evaluated against MCF-7 cell lines [93]. Carrier proteins such as human serum albumin (HSA) and bovine serum albumin (BSA) demonstrated different affinities towards drug interactions, hence, the loading efficacy of TAM and its metabolites with these proteins could be achieved with a loading capacity up to 45–52% [92,94]. Bourassa et al. employed multiple spectroscopic methods and docking studies to investigate the potential conjugation formation of TAM and its two metabolites, 4-Hydroxytamoxifen (4-OHT) and endoxifen, with HSA and BSA [94]. The findings obtained from this study suggested that the conjugations of TAM and its metabolites with both BSA and HSA can result in more stable conjugates than drug-BSA. In another study, Safavi and colleagues employed the high-pressure homogenizer (HPH) and high-speed homogenizer (HSH) to encapsulate TAM inside albumin-bound nanoparticles [95]. The resulting NPs obtained mean diameters from 134.1 ± 5.4 nm to 156.2 ± 2.8 nm, giving rise to a drug loading of 14.2% and 11.6% with HPH and HSH techniques, respectively. The resulting nanoparticles demonstrated equivalent cytotoxicity in BC cell lines, compared to that of free TAM, indicating its promising potential as a drug carrier for TAM. Recent innovations of TAM-loaded-NPs and their advantages in the treatment of BC from 2008–2019, are summarised in Table 3, with respect to their compositions, physiochemical features as well as the claimed advantages.

### 3.4. Other TAM-Loaded Nanoformulations

Aside from the common TAM-nanostructures previously described, attempts have been made to conjugate TAM and its metabolites onto novel and stable nanostructures. Though less commonly fabricated, these systems still offer promising methods to enhance the delivery of TAM to breast tumours, facilitating the therapeutic efficacy of TAM in BC therapy.

Nano-lipid-carriers (NLCs) comprised of both solid and liquid lipids as a core matrix, have been shown to be promising vehicles for the delivery of TAM, owing to their increased solubility, stability, improved permeability and bioavailability, and reduced associated adverse effects [107]. In a recent study conducted by Beh and colleagues [108], erythropoietin-coated nanostructured lipids were employed as TAM carriers (EPO-TAMNLC). Resulting EPO-TAMNLC and TAMNLC showed significant antimammary gland tumor properties, as they induced apoptosis and G_0_/G_1_ cell cycle arrest of LA7, in a dose- and time-dependent manner. By giving an IV dose of 5 mg kg^−1^ body weight, EPO-TAMNLC demonstrated no toxic effects to rats, confirming the safety of this unique DDS. Another novel TAMNLC was developed by How et al. [109], and evaluated for cytotoxicity against human and mouse mammary adenocarcinoma cell lines. Resulting spherical nanosystems achieved an impressive entrapment efficiency of 99.74%. In addition to this optimum drug loading, blank NLC demonstrated relatively low cytotoxic effects, indicating its good biocompatibility. Most importantly, the system demonstrated comparable inhibitory effects compared to free TAM, against both MCF-7 and 4T1 cell lines, confirmed through cell viability studies.

Highly stable nanosuspensions of TAM have been developed using several methods, including pre-milling, magnetic stirring and high pressure homogenisation nano-forms. Surfactants such as Tween 80^®^ (Polysorbate 80) and stabilizer Pluronic F-68^®^ (polaxamer188) have been incorporated into formulations to stabilize resulting suspensions, and to obtain the particle nanosizes of approximately 70 nm. This nanosuspension was reported to be a very promising intravenous solution for the resistance to TAM treatment, due to their extremely small size, and a zeta potential of 8.06 mV which is well within the required range of +30 to −30 mV for nanoparticles suspended in nanosuspension [110].

In another attempt to overcome the hydrophobicity of TAM which negatively affects it systemic transportation, a water-soluble nanoemulsion system was developed by Tagne et al., and evaluated for its antitumour activity [111]. This nanoemulsion demonstrated increased cellular uptake when compared to TAM, owing to the enhanced membrane permeability, which was attributed to its increased net surface negative charge. Most importantly, the TAM nanoemulsion achieved a 20-fold greater cancer cell anti-proliferation and 4-fold increase in cell apoptosis, when tested on HTB-20 BC cell lines [111].

**Figure 5:**
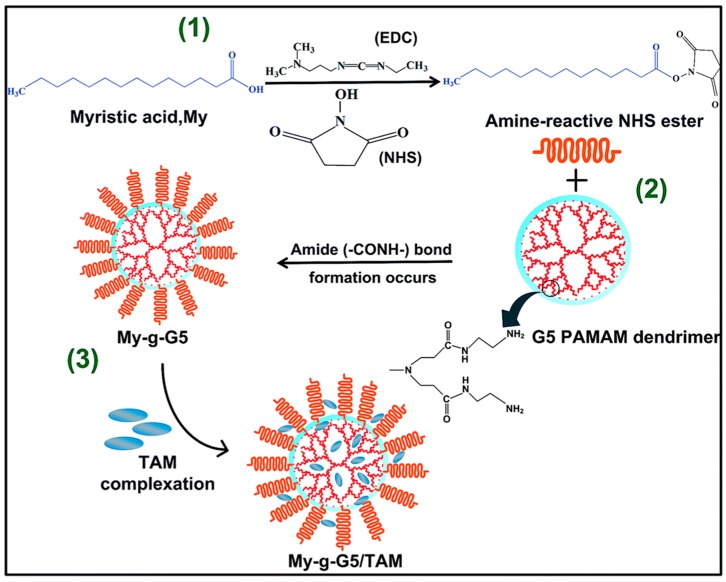
Schematic illustrating the fabrication of My-g-G5/TAM complex: (**1**) The terminal carboxyl groups (–COOH) of myristic acid (My) chains were activated by the addition of EDC/NHS, and magnetically stirred for 12 h in light-sealed condition. (**2**) Resulting solution was added drop-wise into the G5 PAMAM–DMSO solution under N2 atmosphere, and left undisturbed for 24 h at room temperature to form My-g-G5. (**3**) TAM containing solution was slowly added into My-g-G5, resulting in the formation of My-g-G5/TAM complex. Image taken from Matai and Gopinath [113].

Dendrimers are another interesting class of nanocarrier which has received much interest from cancer researchers. Most frequently, PAMAM-(Starburst™), PPI and polylysine-dendrimers are used to fabricate highly branched globular nanopolymeric 3D structures with multiple layers [52,112]. In a recent study, Matai and Gopinath successfully fabricated TAM-entrapped cationic generation-5 polyamido amine (G5 PAMAM) complex, grafted with myristic acid (My), and evaluated for potential anticancer activity against MCF-7 cell lines (Figure 5) [113]. The ability of My-g-G5/TAM to induce apoptosis was confirmed by gene expression studies, confirming this complex is a prospective nanocarrier for TAM [113].

One well-known obstacle associated with cationic macromolecules like dendrimers is their interaction with anionic groups present on biological membranes which may eventually result in their destabilization, disruption and cell lysis [114,115,116]. PAMAM dendrimers are also known to possess concentration and generation dependent cytotoxicity and haemolysis [117,118]. However, it is reported that by using surface functionalisation, the charge-related toxicity of dendrimers can be drastically reduced, greatly enhancing its biocompatibility. One of the most common techniques to mask the positively charged groups on dendrimers’ surface is through acetylation and pegylation [119]. Additionally, the surface of dendrimers can be functionalised with carbohydrate, amino acid, antibody and folic acid residues to abate their net positive charge [119].

Another dendrimer system fabricated from TAM-cyclodextrin (CD) complexes have also exhibited significant anticancer activity against BC with a high loading efficiency of 87.5% and a 91% sustained release of TAM over 120 hours [120]. This complex is believed to be a multifunctional nanomedicine, with simultaneous therapeutic and cytotoxicity studies revealing that TAM-CD complexes significantly inhibit the growth of MCF cells. Moreover, it was able to reduce the TAM-induced hepatotoxicity by almost 30%, owing to surface functionalisation by pegylation [120].

**Figure 6:**
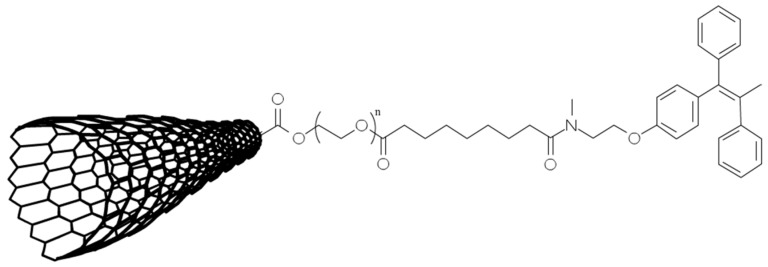
TAM-conjugated-SWCNT: N-desmethyltamoxifen was reacted with carboxylic acid groups (-COOH) present on SWCNT surface. This conjugation was induced by the addition of *N,N*′-diisopropylcarbodiimide (DIC) and 4-dimethylaminopyridine (DMAP) as solvents, in the presence of diisopropylethylamine (DIEA). The resulting solution was filtered and washed with N,N-dimethylformamide and dichloromethane to remove impurities. Image adapted from Oskoueian A., et al. [121].

In a study conducted by Oskoueian et al. (2018), Single-Walled Carbon Nanotube (SWCNT) was fabricated using a chemical vapor method for the conjugation with TAM [121]. As illustrated in Figure 6, by oxidizing SWCNT, PEG was successfully conjugated into SWCNT to give free carboxylic acid and hydroxyl groups as reactive handles (SWCNT-PEG). The SWCNT-PEG was then conjugated with TAM (Figure 6), and the compound was evaluated for cytotoxic concentrations (CC_50_) in MCF-7 cell lines. By linking TAM to functionalised SWCNT, the delivery system enhanced the cytotoxic action against cancer cells of the system up to 2.3 times, compared to free TAM.

Another type of carbon-based nanomaterial frequently employed in the delivery of chemotherapeutic drugs is nanodiamonds (NDs). Due to their small size, high stability, good biocompatibility [122,123,124,125] and the abundance of surface functional groups, researchers can easily construct ND-drug conjugates by generating new ND surface groups, including ether (–COC–), peroxide (–COOR–), carbonyl (–C=O), or carboxyl (–COOH) [126,127]. For example, Landeros-Martínez et al. employed the most recent density functional theory (DFT) studies to investigate a theoretical pathway to the fabrication of ND–TAM complex, to target BC [128]. Following the computational construct of ND-TAM complex, its electronic configuration and hydrogen bonds (HBs) were analysed. The theoretical HBs presented on this complex are C=O⋯H–C, H–O⋯H, and C–H⋯O. These three predicted HBs are attributed to the surface electrostatic interactions between the carrier ND and the drug model TAM, ensuring the stability of ND-TAM complex [128]. Even though this was only a theoretical study with no actual data obtained, it still provides a useful insight into the potential use of ND to deliver TAM.

In another study conducted by Torne et al., TAM-loaded- cyclodextrin (CD) based nanosponges were prepared by conventional inclusion complexation technique, for the oral delivery of TAM [129]. Resulting complexes between β-CD and TAM (TNC) fabricated by freeze-drying method achieved particle sizes of 400 to 600 nm. The plasma concentrations TAM released from oral TNC were significantly higher than that of plain TAM at the same dose. In addition to this, the mean absolute bioavailability of TNC achieved 1.45-fold higher than native TAM. Furthermore, cytotoxicity study against MCF-7 cell line revealed that TNC showed greater inhibition of cell proliferation after 24 and 48 h of incubation, compared to free TAM. However, it is not explained by the authors the exact mechanism by which TNC demonstrated enhanced toxicity against tumours, as compared to native TAM. Regardless of this, the study presented a very promising nanocarrier system for the effective delivery of TAM in BC therapy, with enhanced pharmacokinetic parameters of loaded TAM as compared to free TAM.

Similarly, in a study performed by Elnaggar and colleagues, self-nanoemulsifying drug delivery systems (SNEDDS) containing TAM were prepared to circumvent its low hydrophilic solubility [130]. Various ingredients, their compositions and surfactants were pre-screened for their bioactivities; and were examined for different parameters, including size, morphology, robustness to dilution, and drug release. The selected and optimized formulation contained TAM (1.6%), maisine 35–1 (16.4%), caproyl 90 (32.8%), cremophor RH40 (32.8%) and propylene glycol (16.4%). Resulting SNEDDS were spherical in shape, with an obtained mean globule size of 150 nm. TAM released from selected SNEDDS was significantly higher than other SNEDDS and plain TAM suspensions, indicating a potential of improved oral efficacy of free TAM.

Additionally, various nanotechnology-related strategies and advanced materials have also been developed to enhance the chemo-preventative ability of TAM in BC therapy. For example, Ballerini and colleagues successfully employed a novel nanochannel delivery system (nDS) for the long-term delivery and sustained release of TAM in mammary tissues [131]. PEG400 was used as a solubilizer to overcome the low hydrophilic solubility of incorporated TAM. A sustained and steady level of TAM was achieved in mammary tissues over several months (>2 months), maximizing its therapeutic index and chemo-preventative ability. In addition to this, by placing the nDS adjacent to the mammary gland, the risk of whole-body exposure and off-target secondary effects will be significantly reduced, enhancing treatment efficacy and patient compliance of resulting nanosystem.

Despite the aforementioned advantages of TAM-loaded nanosystems, there are still some major challenges to overcome derived from the bio-degradability and bio-compatibility of nanomaterials used for the delivery of TAM [93]. Moreover, the lack of clinical evidence and translatability between in vitro and in vivo conditions have hindered the use of nanomaterials as delivery systems for TAM, requiring more innovative research attempts into this new and exciting area.

### 3.5. TAM as a Targeting Vector

**Figure 7:**
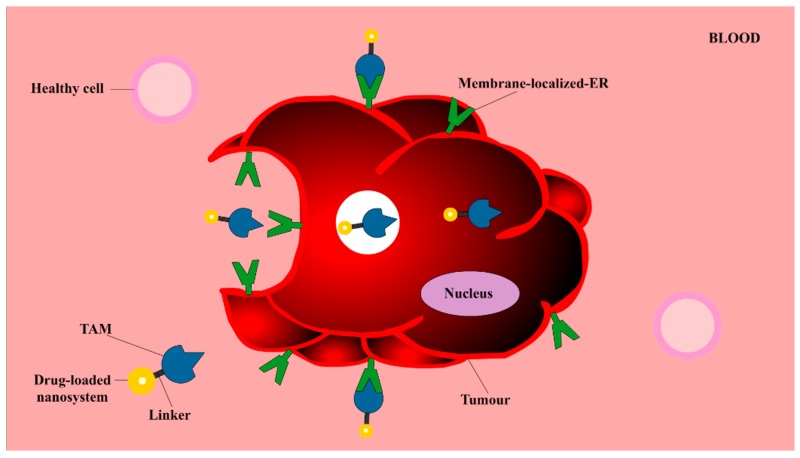
The internalization of TAM-guided nanosystems into breast tumour: TAM is employed as an active/targeting vector due to its ability to recognize and bind specifically to ERs locating on the membrane of tumours, namely membrane-localized-ER. By conjugating TAM at the distal end of various nanosystems containing other therapeutic materials (including different drugs), selective delivery and receptor-mediated cellular internalisation of incorporated materials can be initiated [132,133,134]. Image adapted from Barclay et al. [134].

It is undeniable that TAM-loaded nanosystems are promising solutions to the issue of dose-dependent toxicity of TAM, owing to the significant lower concentration of TAM delivered selectively to a target site. Additionally, TAM can also be employed as a *guiding vector*, directing the delivery of nanosystems containing other therapeutic agents to cancer cells. The concept of using TAM as a targeting ligand in addition to using it as an antitumour agent has emerged as a novel strategy which utilises the strong binding affinities of TAM with key cancer cell targets [132,133]. Acting as a targeting vector, low concentrations of TAM can be used to guide the delivery of other therapeutic drugs to ER+ BC cells, utilising the anticancer properties and targetability of TAM, whilst decreasing its dose-dependent-toxicity. The phenomena in which TAM can be used as a targeting vector, is illustrated in Figure 7. TAM-guided nanosystems can actively recognise and bind selectively to membrane-localized-ER, initiating receptor-mediated cellular internalisation processes [132,133,134]. By using TAM as a targeting vector, nanosystems can achieve dual functions, allowing TAM to act synergistically with other therapeutic agents to kill cancer cells and to induce internalisation of the system into ER+ cancer cells.

**Figure 8:**
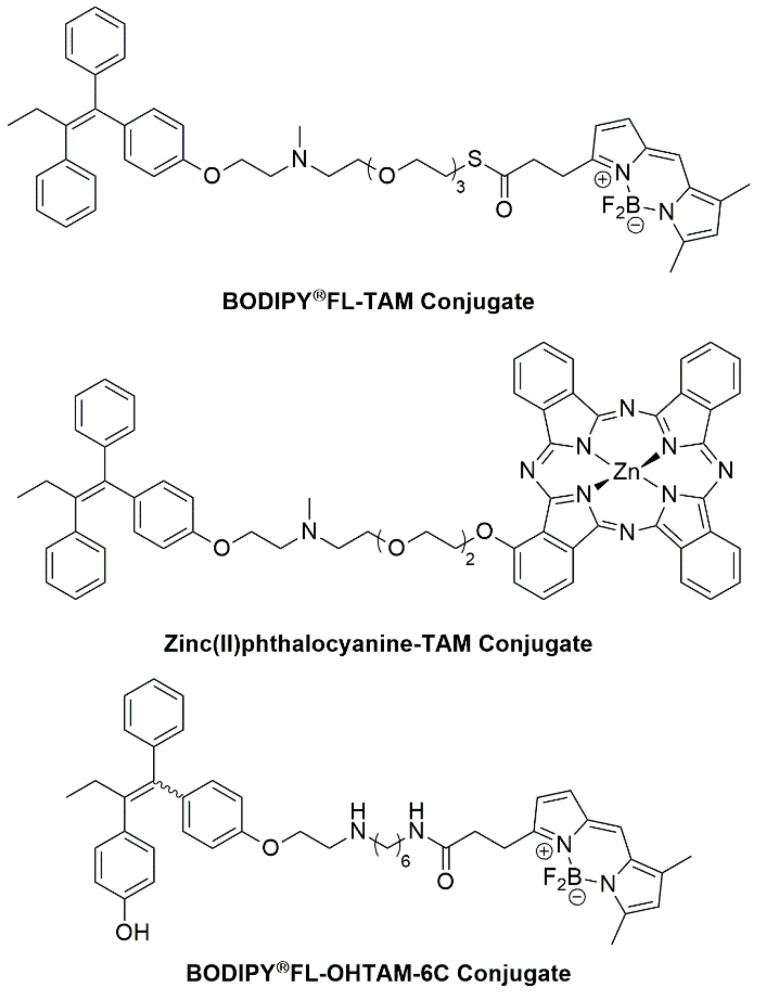
Examples of TAM conjugates [135,136,137].

The ability of TAM to be a targeting agent was first proven by its ability to deliver fluorescent dyes into tumours, through the conjugation of TAM with various fluorophores, as seen in Figure 8. In a study conducted by Ho et al. [135], a novel ER+ targeted fluorescent probe was prepared which obviated the need for costly labelling of cells with specific antibodies. The TAM-BODIPY^®^FL probe employs a tetraethylene glycol (TEG) unit as the linker between targeting vector (TAM) and fluorophore, as demonstrated in Figure 8, and exhibited selective binding to ER+ and ER- cell lines [135]. Even though this probe showed no affinity for TAM resistant BC cell lines, it still offered a useful insight into the biology of cancer cells.

Zhang and colleagues also used the non-toxic amphiphilic spacer TEG to conjugate *N*-desmethyltamoxifen and a Zinc(II) phthalocyanine moiety, as indicated in Figure 8. The new conjugate was found to have strong binding affinity to ERs, exerting both BC photodynamic and hormone therapy [136]. The resulting conjugation possesses promising biocompatibility, exhibiting dual photo-hormone therapy (PHT) for BC. A higher cytotoxicity profile with 50% cancer cell killing effect was obtained when this conjugation was evaluated on MCF-7 cell lines, suggesting potent cancer apoptosis properties [136].

In another study by Rickert et al. [137], an analogue of TAM with a 1,6-diaminohexane linker, known as OHT-6C, was successfully synthesized and conjugated into the BODIPY^®^FL fluorophore (Figure 8). Fluorescence microscopy was used to investigate the cellular uptake and cytoplasmic localization of this fluorescent conjugate, on the basic of brightness and potency quality. The BODIPY^®^FL-labelled-OHT-6C compound was shown to be effective at inhibiting proliferation in both TAM-sensitive and TAM-resistant cell lines.

In addition to tagging TAM with fluorophores, TAM can also act as a vector to deliver other nanosystems containing therapeutic and diagnostic cargo. For example, liposomes derived from phospholipids using a lipid film hydration technique, has been employed as nanovehicles to deliver DOX to breast tumours [132]. TAM was firstly incorporated into the lipid bilayers of liposomes, following by the addition of DOX solution into TAM liposomal dispersion at 54 °C for 45 min. The system produced a positive result as observed with an increased and faster uptake of liposomal DOX into tumours and the cancer cell viability of MCF-7 cell lines was reduced [132]. Even though the authors originally concluded that TAM acted as a targeting ligand for liposomal DOX was evidenced with cellular uptake studies, it is unclear from this article how TAM is acting as a targeting vector on the polar surface of the phospholipid when they the author incorporated TAM into the lipid bilayer of liposomes instead. It is important to characterise the surface of liposomes to ensure TAM molecules are situated in desired locations to exercise their ability of recognise and bind to targets. The information regarding the conditions by which tumour cells were incubated and prepared for uptake study, in term of temperature, and the presence of metabolic inhibitors to block other non-specific uptake pathways, were not provided in this study. It is therefore possible that passive targeting due to the nano sizes of the system may account for some uptake efficacy.

In another study conducted by Dreaden et al., TAM was employed to selectively target and deliver plasmonic gold nanoparticles to ER+ BC cells [133]. TAM molecules were attached to the gold particles via a PEG linkage. A 2.7-fold enhanced drug potency was observed when tested on MCF-7 lines. Time-dependent dose-response data and estrogen competition studies indicated this system induced cellular uptake of TAM via receptor membrane localised estrogen receptor-mediated transportation, instead of passive diffusion which facilitates the movement of the free drug.

As demonstrated with the aforementioned studies, conjugating low concentrations of TAM to diagnostic agents such as fluorophores, or nanoformulations containing therapeutic agents such as DOX, often increases cellular uptake and cytoplasmic localization can be quantified more easily. By using TAM as a guiding vector, not only can the undesired off-target effects be overcome but the therapeutic power of TAM can be utilised.

## 4. Authors’ Opinion on the Future of Nanoformulated TAM

TAM has cemented its therapeutic value for BC compared to other treatment options, owing to its effectiveness and affordability. The initial antihormone concept for which TAM was used to treat BC has pioneered new treatments for various hormone-driven diseases. Like all anticancer drugs, there are still concerns about the adverse effects of TAM, such as the risk of developing endometrial cancer in post-menopausal women. Reducing the dose of TAM offers a promising strategy to overcome the side effects associated with conventional oral doses. By employing novel and effective TAM-containing nanomedicines as an alternative to chemotherapy, the many toxic effects caused by chemotherapy will be greatly minimized. Nanodrug delivery systems are capable of delivering lower concentrations of TAM to tumours over a prolonged period of time, limiting its dose-dependent toxicity. Additionally, the incorporation of TAM into nanosystems, low concentrations of TAM can also be employed as a targeting ligand, specifically to direct the delivery of more potent diagnostic fluorophores and other anticancer agents to solid breast tumours, obtaining higher accuracy and minimal off-target toxicity.

Despite recent advances of novel TAM nanoformulations, they have not completed the regulatory assessments required to proceed with clinical development. This may be explained by the fact that most of the nanoformulated products have demonstrated reduced toxicity, rather than improved efficacy compared to conventional TAM, which is a key requirement to gain regulatory approval [138]. In addition to this, as discussed in the scope of this review, various TAM-enabled nanosystems have shown promising results in both reduced toxicity and improved efficacy, however the lack of translatability between in vitro and in vivo conditions remain a significant challenge. Nanoformulated TAM which initially demonstrates superior efficacy in cancer cell lines may not retain efficacy in animal models [139]. Therefore, more effort is required to design high-throughput techniques to improve the predictive nature of in vitro methodologies compared to in vivo conditions.

Another challenge of nanoformulated TAM arises from the compatibility of nanomaterials used as TAM carriers with biological systems. Dendrimers, carbon- or silica-based nanomaterials can interact with membrane phospholipids on the surface of red blood cells (RBCs), resulting in haemolysis, or the rupture of RBCs [117,118,140]. Nanomaterials have also been reported to increase reactive oxygen species (ROS) generation which induces oxidative stress [140,141,142,143]. Therefore, it is crucial to understand how different cell types will react to nanomaterial exposure to allow for a more accurate assessment of the risks vs. benefits of nanomaterials. Once this knowledge gap is addressed, researchers can design improved formulation strategies to protect healthy cells from potential toxicity issues associated with nanomaterials.

The toxicity of nanomaterials can be reduced through surface engineering, or with pegylation [119,141]. The PEG coating is frequently used to mask the surface net positive charge of nanomaterials, preventing their interaction with negatively charged cell membranes [119]. Additionally, pegylation can help shield nanoparticles from aggregation, opsonization and phagocytosis, improving their stability in biological fluids and enhancing the circulation times of these systems [144,145,146,147]. Indeed, the very first FDA-approved nanomedicine is Doxil; a pegylated-DOX-liposome [55]. As summarized in Table 1, Table 2 and Table 3, various TAM-containing formulations have introduced PEG into their systems to enhance the delivery of TAM to breast tumours. For example, in cases of liposomes formulated with pegylated lipids, or micelles consisting of pegylated-coblock polymers, pegylation has been found to increase circulation stability and enhance the resistance to clearance and protein adsorption on the surface of NPs.

Despite striking benefits of surface engineering of nanomaterials with PEG, the role of pegylation in nanoformulated systems still remains a debate. Firstly, it is acknowledged that pegylation cannot completely resist interactions of nanoformulated systems with blood components, as the protein adsorption of pegylated nanoformulations is only reduced, not eliminated [148,149]. Secondly, potential PEG immunological issues or the phenomenon in which pegylated nonhuman enzymes trigger the anti-PEG antibody (Ab) responses; have caused considerable concern regarding the use of pegylation onto nano DDSs [150,151,152,153,154,155,156]. The anti-PEG Ab responses may hinder the efficacy of nanosystems in drug delivery fields, as repeated administrations of the carrier system exhibits shorter drug half-life, more rapid clearance, or the loss of prolonged blood circulation which is one of the key benefits of nanoformulated systems [153,154,155,156,157]. Although the anti-PEG Ab responses have not been reported in nanoformulated TAM, it is important to take these potential phenomena into account during the fabrication of TAM-enabled nanosystems to avoid immunological issues. Finally, the potential issue of pegylation in nanoformulated TAM arises when TAM is used as a targeting vector, as discussed in Section 3.5. To act as a targeting vector, TAM molecules have to be conjugated at the distal end of nanosystems. However, if these carriers are to be further pegylated, the PEG coating will mask the presence of TAM molecules, preventing them from “seeing” and binding to ER, leading to a negative receptor/ligand recognition phenomenon [157]. The question of whether or not pegylation should be used in formulated TAM systems remains a formidable challenge for scientists, and it is an important factor to be considered in the future design of TAM-nanoenabled formulations.

An emerging trend in the field of nanomedicines is the design of stimuli-responsive DDSs [158]. Within these DDSs, the individual variability, sustained release profile and targeted permeability of incorporated drugs can be controlled in a sophisticated manner, in response to external stimulus include temperature, light, or magnetic fields; and intrinsic stimulus of pathological sites, such as pH, temperature and redox status [158,159]. For example, Yang and colleagues prepared a biocompatible nano-photosensitizers (PS) from chlorin e6 (Ce6) modified HAS and TAM (HSA–Ce6/TAM) [160]. The nanocomplexes remained stable under neutral pH, and would be dissociated separately into HSA–Ce6 and TAM molecules under the acidic tumour microenvironment, due to the pH responsive transition of TAM from hydrophobic to hydrophilic. HSA–Ce6/TAM nanoparticles not only demonstrated prolonged systemic circulation due to their stability at neutral pH, they also efficiently attenuated the tumour hypoxia status, owing to the ability of TAM to reduce the oxygen consumption of cancer cells [160]. In another study, temperature-sensitive hydrogels containing TAM and triblock-copolymers PLGA-PEG-PLGA of 120 nm were generated, with the ability to transform TAM solution into hydrogels at room temperature [161]. Long-term release of TAM from TAM-gel was sustained for more than 400 h, with the half-life of released TAM achieved of 194.41 ± 12.60 h. Following administration, TAM-gel significantly inhibited the long-term uptake of radionuclide probes (^18^F-fluoroestradiol or ^18^F-fluorodeoxyglucose), and intrahepatic growth of MCF-7 in rats’ livers [161]. By incorporating TAM into these carriers, dual/multi-responsive DDSs will be fabricated, utilising the therapeutic potentials of multifunctional drug TAM. Owing to the combination of various functions into one system, this lays a foundation for a new generation of intelligent release-on-demand nanoformulated TAM to be devised.

As the use of nanomedicines as an alternative treatment for conventional chemotherapy is on the rise, with estimated global revenue of nanodrugs at $178 billion in 2019 [55], it is reasonable to conclude that research efforts into new nanoformulated TAM are also increasing. However, as they are still in early stages of development, expectations regarding clinical outcomes need to be realistic. In addition to the regulatory issues, the large-scale production of nanoenabled-TAM formulations is another obstacle, due to the high cost of materials, experts and preparation processes required. Further research and clinical trials are required to establish the most effective strategies to utilise the targetability of the versatile TAM, especially when their benefits clearly outweigh the risks. Through these strategies, the old yet remarkable drug TAM may be given a new and more powerful life and provide us with enhanced tools in the fight against BC.

## Figures and Tables

**Table 1:** TAM-loaded-liposomes from 2008–2019 and their applications in BC.

Composition *^a^*	Physiochemical Properties *^b^*	Advantages/Remarks	Ref
TAM, SPC, CH, span 20	MD = 203.5 ± 19.5 nm PI = 0.442 ± 0.03	Good vesicular distribution DL = 53.6% 4% of the drug loss over 5 weeks 95% of drug was released in 30 h	[59]
TAM, PEG-PEI	PS < 270 nm ZP = 40 mV	6.7- to 7.9-fold increase in cytotoxicity 86% inhibition of tumour growth in BT474 tumour bearing mice No induction of skin/organ injury	[60]
TAM, CH and lipid (DMPC and/or DSPC)	PS = 482 + 0.013 nm to 887 ± 0.336 nm Smooth surface Multilamellar, disc shaped ZP = −37.2 to −41.2 mV	Good stability Sustained drug release up to 10 d after initial burst release	[62]
TAM, Saturated SPC, phospholipid GmbH	N/A	Significant dose-related reduction in cell viability (MCF-7 cell line)	[63]
TAM, GEM, DPPC, CH, DMPG and DSPE-MPEG 2000*^c^*	PS = 150–200 nm Surface charge = 50 mV and −30 mV	Strong cell interaction after 6 h	[61]
TAM, DAU, EPC, PEG2000-DSPE, CH, SRB	EE = 95% (DAU) and 90% (TAM) PS = 100 nm	Promising effects in eliminating both BC cells and cancer stem cells	[64]
TAM, Imatinib, DPPC, MPPC	EE > 70% 80% drug release with temperature-responsive behaviour (within 30 min above transition temperature 34.9 °C).	Synergistic growth inhibition against MCF-7 and MDA-MB-231 breast cancer cells	[65]

*^a^* SPC = soya phosphatidylcholine, CH = cholesterol, PEI = polyethylenimine, DMPC = dimyristoyl phosphatidylcholine, DSPC = distearoyl phosphatidylcholine, GEM = gemcitabine, DPPC = 1,2-dipalmitoyl-s*n*-glycero-3-phosphocholine, DMPG = dimyristoyl phosphatidyglycerol, DSPE-MPEG-2000 = *N*-(Carbonylmethoxypoleythylene glycol-2000)-1,2,-diastearoyl-s*n*-glycero-3-phosphoethanolamine, DAU = daunorubicin, EPC = egg phosphatidylcholine, SRB = sulforhodamine B, MPPC = monopalmtoyl-2-hydroxy-s*n*-glycero-3-phosphocholine. *^b^* MD = mean diameter, PI = polydispersity index, PS = particle size, ZP = zeta-potential, EE = encapsulation efficiency, DL = drug loading. *^c^* Liposomal formulations were prepared by the TLE technique and the extrusion process.

**Table 2:** TAM-loaded-micelles from 2008–2019 and their applications in BC.

Composition *^a^*	Physiochemical Properties *^b^*	Advantages/Remarks	Ref
TAM, PEG5000-PE	PS < 200 nm DL: 1:5 ratio drug/polymer w/w (20 wt % of TAM)	75% TAM retained after 48 h Increased TAM accumulation into C57BL/6J tumour bearing mice	[69,70]
TAM, PLGA–PEG	Spherical PS = 76.4 ± 2.1 nm ZP = −4.89 mV (neutral) EE = 60.86 ± 3.21%	Controlled release profile, the cytotoxic potential of TAM against MCF-7 cell lines was substantially enhanced	[71]
TAM, CS, TS	Spherical PS < 200 nm	pH-dependant release profile Increased stability in GIT Increased oral bioavailability	[72]
TAM, Palmitic acid, CS	PS = 83.71 ± 0.15 nm ZP = +37 ± 0.14 mV EE = 93.76 ± 0.40% DL = 10.24 ± 0.21%	Controlled release profile Enhanced antitumour activity on MCF-7 cells IV formulation has better haemo-compatibility 2.5-fold enhancement of half-life and 1.7-fold reduction of clearance	[73]
TAM, CS, PLGA	PS = 81.48 nm PDI = 0.209 ZP = +19.27 ± 4.34 mV	Controlled release at near to neutral pH Enhanced efficacy and cellular uptake Increased dermal/epidermal bioavailability	[74]

*^a^* PS = particle size, PLGA = poly(latic-co-glycolic acid), CS = carboxymethyl chitosan, TS = α-tcopherol succinate. *^b^* PDI = polydispersity index.

**Table 3:** TAM-loaded-nanoparticles from 2008–2019 and their applications in BC therapy.

Composition *^a^*	Physiochemical Properties *^b^*	Advantages/Remarks	Ref
*Examples of Polymeric NPs*			
TAM, LMW CMC, TS	LD = 8.08 ± 0.98%	1.9 fold increases in AUC_0-72h_, suggesting superior safety profile compared to free TAM	[76]
TAM, PLGA	Smooth surface PS = 250–380 nm Maximal DL = 27.16 ± 2.08%	Sustained drug release pattern up to 60 days Enhanced antitumour efficacy against MCF-7	[77]
TAM, Poly(d,l-Lactide)	NPs prepared by emulsification solvent diffusion method PS = 271.4 nm ZP = 34 mV EE = 76.4%	Biodegradable nanoparticles Improved antitumor activity against MCF-7	[78]
TAM, NIPAAM, VP, PEG-DA	NPs prepared by gamma irradiation polymerization Core-shell structure Spherical or elliptical shaped Smooth surface Size distribution = 49.89 nm (SD ±1.82)	Greater inhibitory effect on MCF-7 cells compared to free TAM	[96]
TAM, CS, Pluronic	Spherical shaped with positive charge MD = 150–300 nm EE = 8mg/mL TAM	Good blood compatibility Bare particles are nontoxic to cells	[97]
TAM, CS	MD = 100–150 nm DL = 28%	pH-dependent release behaviour Faster and higher TAM release at pH 6 (43 ± 0.45%) and pH 4 (68 ± 0.34%), slower release at pH 7.4 (22 ± 0.21%) Increased tumour uptake of TAM in MCF-7 cell-line Induced caspase-dependent apoptosis	[98]
TAM, PAA, CH	NPs prepared by electrospray technique PS <500 nm Spherical shaped DL = 40%	Higher dose-dependent cytotoxicity than free TAM Blank NPs were nontoxic against MCF-7 cell lines	[99]
TAM, PLGA, AuNPs	EE = 30%	Highly effective against MCF-7 cell lines	[100]
TAM, Guar gum (GG)	TAM, GG was crosslinked with glutaraldehyde NPs prepared by oil in water (o/w) emulsion polymer cross-linking method DL = 15% PS = 200–300 nm	Maximum uptake and retention of TAM-loaded- NPs in the mammary gland observed	[101]
TAM, PCL (MW ∼ 15, 000), Pluronic^®^ F-68, F-108, PEO, PPO	NPs prepared by solvent displacement Spherical shaped ZP ∼25 mV PS = 200–300 nm	Increased tumour concentrations in MCF-7 cells Longer retention times within tumours Pluronics (both F-68 and F-108), preferential concentration within the tumour mass via enhanced permeation and retention pathway, and controlled release	[102]
TAM, QT, PLGA	PS = 185.3 ± 1.20 nm PDI = 0.184 ± 0.004 EE = 67.16 ± 1.24% for TAM, 68.60 ± 1.58% for QT	Enhanced cellular uptake, cytotoxicity and nuclear co-localisation in MCF-7 cells No measurable hepatotoxicity/oxidative stress	[103]
*Example of Polymer-lipid Hybrid NPs*			
TAM, Tween 80, CS, Lecithin	NPs prepared by modified solvent emulsification-evaporation method PS = 169.66 ± 4.84 nm	Prolonged in vitro release profile Enhanced oral bioavailability Increased antitumor efficacy in DMBA-induced BC model	[104]
*Examples of protein base-based NPs*			
TAM, SA, thiolated alginate (alginate-cysteine conjugate)	MD = 446, 430 and 498 nm ZP = −37 ± 9, −26 ± 7, and −7.2 ± 0.3 mV	Drug loading affected particle size significantly	[93]
TAM, 4-OHT, Endoxifen, HSA, BSA	EE = 45–52% for each drug-protein conjugate Free binding energy of hydrogen bonding was 11.79 to −11.25 Kcal/mol (drug-HSA) and −13.79 to −12.72 Kcal/mol (drug-BSA conjugates)	High loading of TAM and metabolites HSA and HBA are promising carriers for the transportation of TAM, 4-OHT and Endoxifen	[94]
TAM, albumin	NPs prepared by HPH and HSH DL = 14.2 ± 1.9% (HPH); DL = 11.6 ± 2.3% (HSH)	Better particle homogeneity Decreased BT474 cell viability	[95]
*Examples of SLNs*			
TAM, Hydrogenated palm oil, Hydrogenated soybean lecithin	NPs prepared by HPH PS = 251.65 ± 33.02 nm ZP = +10.16 ± 0.22	Induced apoptosis in the MCF-7 and MDA-MB231 BC cells Lower hepatotoxic effects	[79]
TAM, Stearic acid 5%, Tween 80 2.5%	NPs prepared by HH Circular shaped PS = 2283 ± 1.88 nm PDI = 0.298 and Tam-SLNs	Good homogeneity, narrow size distribution Bypass TAM resistance by miRNA downregulation	[80]
*Example of Liquid Crystalline NPs*			
TAM, Glyceryl monooleate, Phytantriol, Oleic acid	Hexagonal PS = 154.93–235.76 nm PDI = 0.18–0.24 nm EE = 79–81%	TAM-LCNPs more toxic against MCF-7 cells compared to TAM 5 to 7x oral bioavailability enhancement Reduction in tumour burden Lower hepatotoxicity	[81]
*Examples of Precious Metal NPs*			
TAM, Adenia hondala tuber extract, CS, AgNPs	MD = 60–140 nm	pH dependent Dose-dependent cell death	[82]
TAM, Ag^+^, AgNPs	PS = 1–28 nm	Both the combination of Ag ion and TAM, and Ag NPs-TAM, demonstrated induced cytotoxic to TAM-resistance T47D cell line	[105]
*Examples of Magnetic NPs*			
TAM, Tyrosin, Fe_3_O_4_	ZP = − 12.8 mV PS = 22.19 ± 3.58 nm DL = 11.34 ± 0.09% EE = 51.21 ± 0.41%	Biocompatible Promising anticancer activity Suitable carriers for hydrophobic drugs	[83]
TAM, Fe_3_O_4_, APS-PEG-BrAc	PS = 40 nm DL = 49.1%	Sustained TAM release from NPs Inhibits MCF-7 cell growth	[106]

*^a^* LMW CMC = low molecular weight carboxymethyl CS, NIPAAM = nisopropylacrylamide, VP = *N*-vinyl-2-pyrrolidone, PEG-DA = poly(ethylene glycol) diacrylate, PAA = poly(amidoamine), NP = nanoparticle, PCL = poly(ε-caprolactone), PEO = poly(ethylene oxide), PPO = poly(propylene oxide), QT = quercetin, SA = serum albumin, APS-PEG-BrAc = bromoacetyl-terminal PEG silane. *^b^* HPH = high pressure homogenization, HSH = high speed homogenization, HH = hot homogenization.
